# Supplementary material for: A simplified minimodel of visual cortical neurons
Source: Nat Commun. 2025 Jul 1;16:5724. doi: 10.1038/s41467-025-61171-9 (PMC12219398; doi:10.1038/s41467-025-61171-9)
Supplement: Supplementary file 1 — Supplementary Information [file 41467_2025_61171_MOESM1_ESM.pdf]

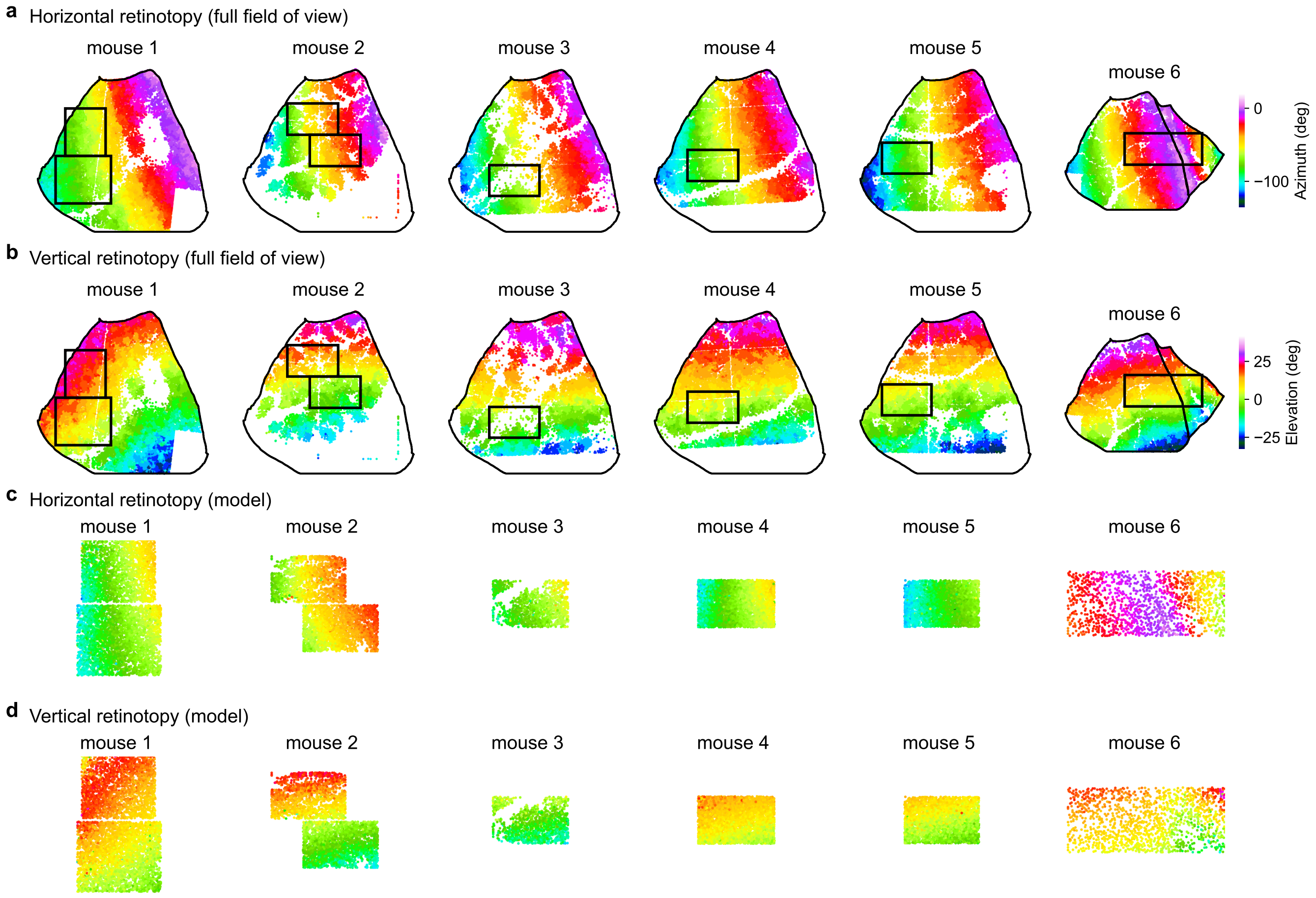

Supplementary Fig. 1: Recording locations and retinotopic maps in mouse V1. a-b, Retinotopic maps of V1 neurons in six mice. Each neuron recorded is colored by its preferred visual angle in azimuth (a) and elevation (b). The black rectangles represent the position of the 30Hz recording during which the $>$30,000 natural images were presented. **c-d,** Each neuron in the zoomed-in recording colored by the center-of-mass of its readout weights ($\mathbf{w}_x$ and $\mathbf{w}_y$) from the 16-320 model (Fig. 2).

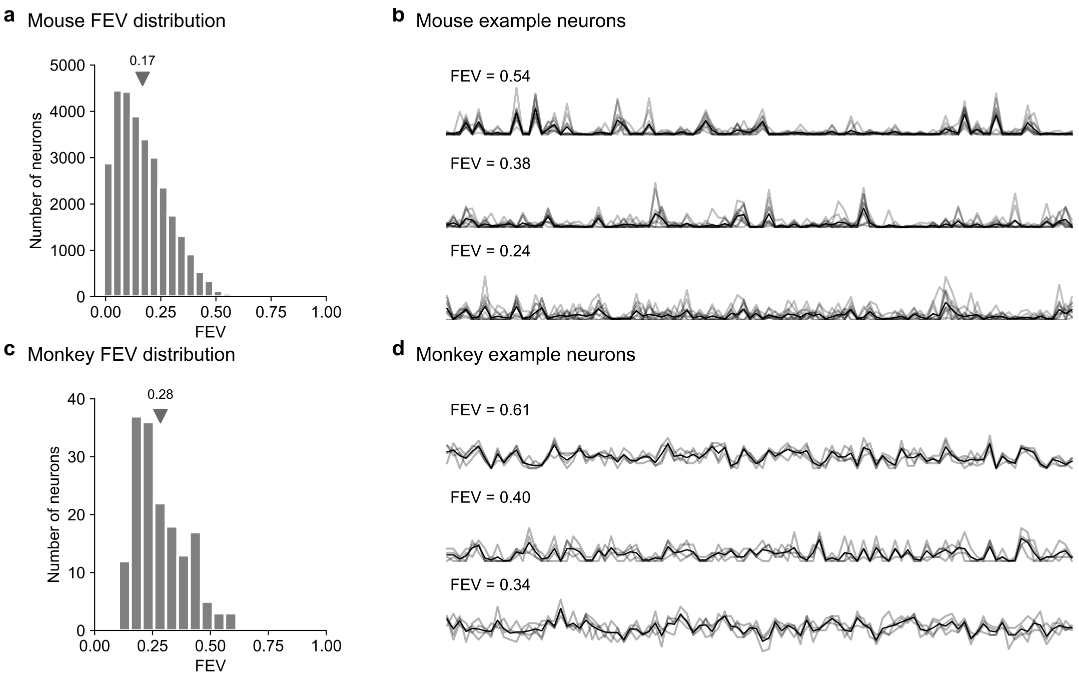

Supplementary Fig. 2: Fraction of explainable variance (FEV) of mouse and monkey neurons. a, Fraction of explainable variance (FEV) distribution of all neurons recorded from mouse V1 (N=6 mice with 29,608 neurons) and the mean across all neurons (triangle). **b,** Responses of three example neurons to ten repeats of the same natural images (gray lines), and the mean response across all repeats (black line). **c-d,** Same as (a-b) for all monkey V1 neurons. Note that this only includes the neurons with FEV $>$ 0.15, which were preselected in the Cadena dataset.

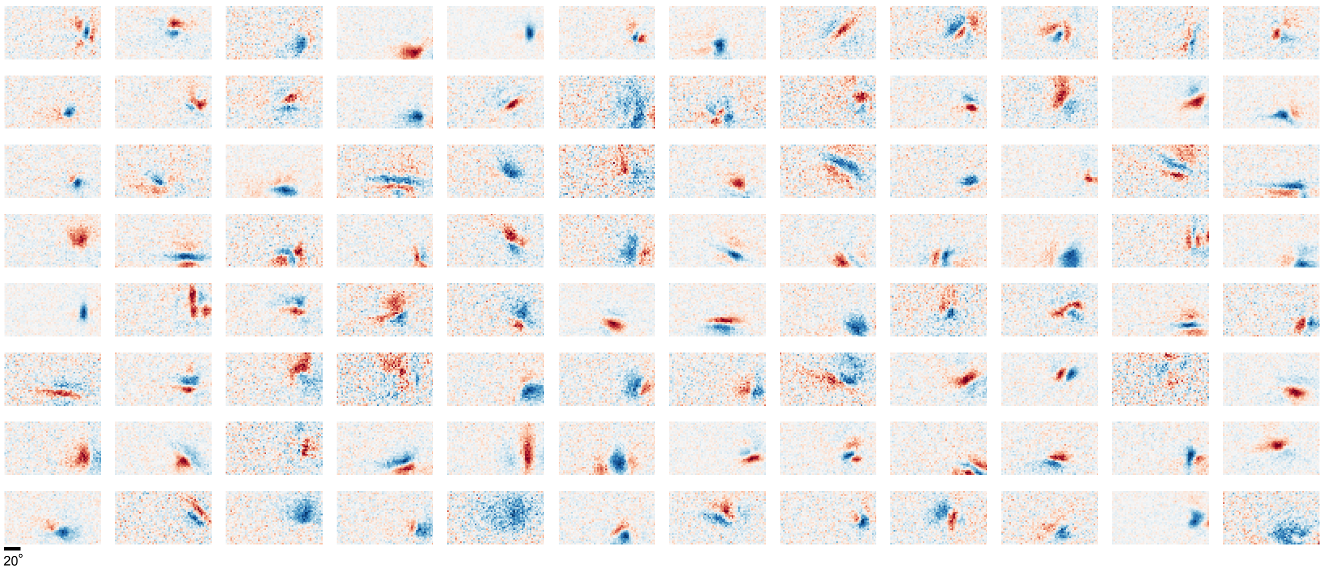

Supplementary Fig. 3: Linear receptive fields of randomly-chosen neurons. Each rectangle is the linear RF of a single randomly-selected neuron with FEV $>$ 0.15 (the criterion for inclusion in the study) from an example mouse. For estimation, images were downsampled by a factor of two in each dimension, but no other constraints of regularization or smoothness were imposed in the estimation.

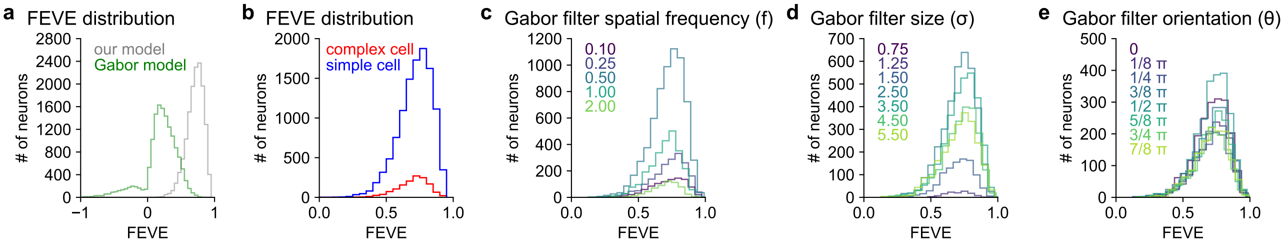

Supplementary Fig. 4: Relationship between FEVE and single-neuron properties. **a,** Comparison of FEVE distributions between the Gabor model (see Methods) and our model (N=6 mice with 14,504 neurons). Only neurons with positive FEVE values in the Gabor model were included in subsequent analyses (N=6 mice with 12,637 neurons). (**b-e**). **b,** FEVE distributions for complex cells (red) and simple cells (blue). **c-e,** FEVE distributions for neurons associated with different Gabor filter (c) spatial frequencies, (d) sizes, and (e) orientations.

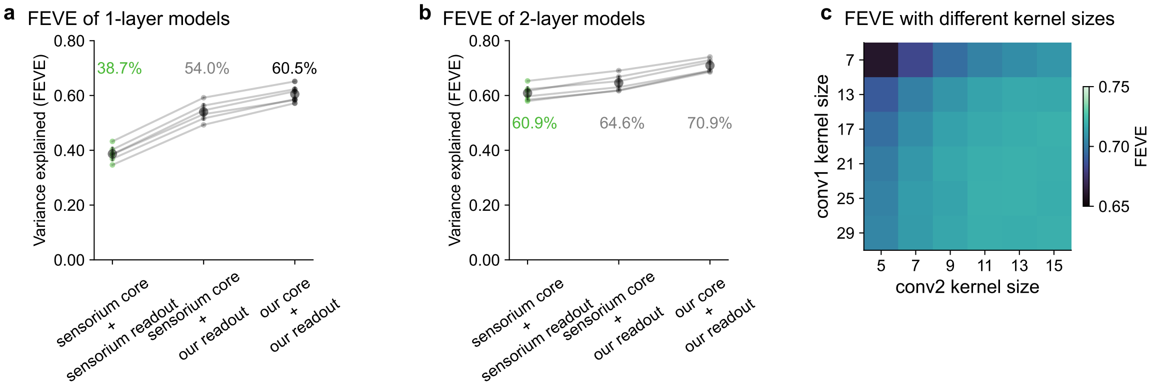

Supplementary Fig. 5: Effect of the model architecture on performance. **a,** Performance of the 1-layer model with different core and readout configurations (N=6 mice). **b,** Performance of the 2-layer model with different core and readout configurations. **c,** Performance of the 2-layer model with varying conv1 and conv2 kernel sizes.

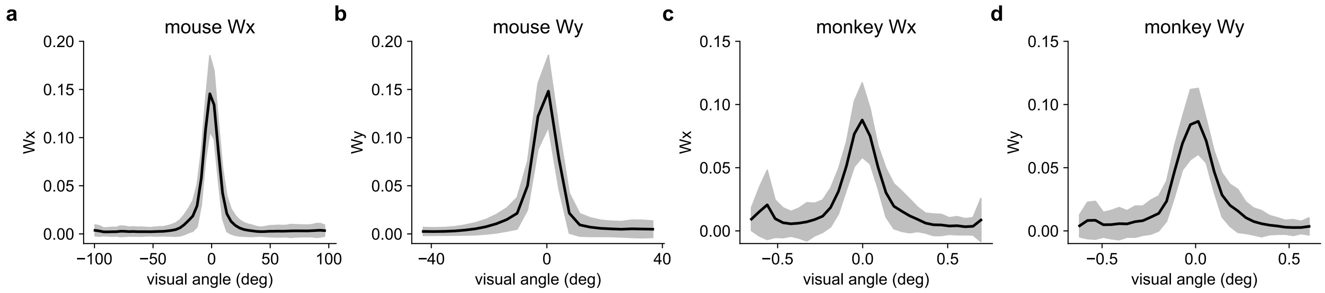

Supplementary Fig. 6: Localized weights $\mathbf{w}_x$ and $\mathbf{w}_y$ learned by the model. **a-b,** Spatial tuning curves of mouse V1 neurons (N=14,504 neurons) derived from the model weights $\mathbf{w}_x$ and $\mathbf{w}_y$. We aligned the $\mathbf{w}_x$ and $\mathbf{w}_y$ from different neurons so that their peak positions were at 0 degrees, and computed the mean across all neurons (black). Error bars represent the standard deviation. **c-d,** Same as **a-b** for all monkey V1 neurons (N=166 neurons).

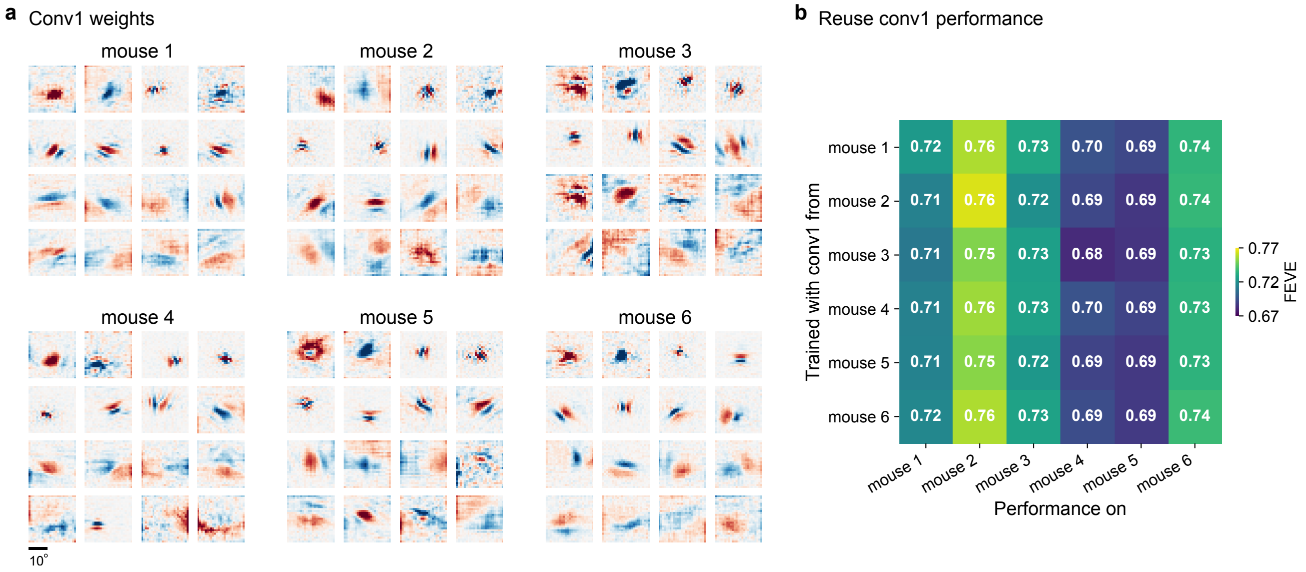

Supplementary Fig. 7: Cross-validation of conv1 weights in minimodels across mice. a, Conv1 weights from the 16-320 encoding model of all six mice. **b,** The performance of minimodels when trained using conv1 weights from one mouse but tested on another. For each mouse, we randomly selected 100 neurons and trained minimodels using the conv1 weights from the 16-320 full encoding model of one of the six mice. Each row represents the performance of minimodels for all six mice, utilizing conv1 weights from one of the mice. Performance is measured in terms of the FEVE.

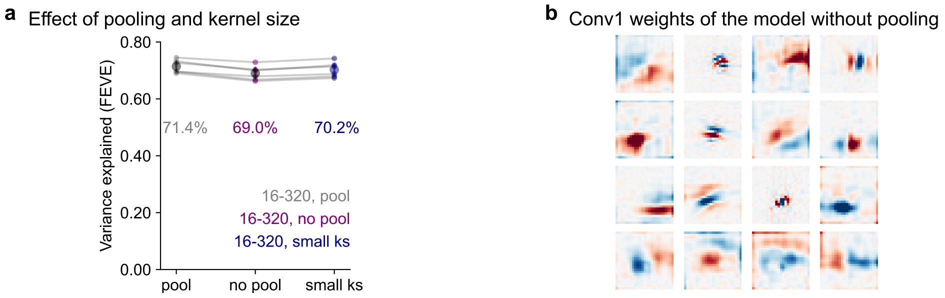

Supplementary Fig. 8: Impact of max pooling and conv1 kernel size on model performance. a, Performance comparison of the 16-320 model with and without max pooling after the first layer, and with max pooling and smaller conv1 kernel sizes (9x9 vs 25x25) (N=6 mice). **b,** Visualization of conv1 weights from the 16-320 model without max pooling.

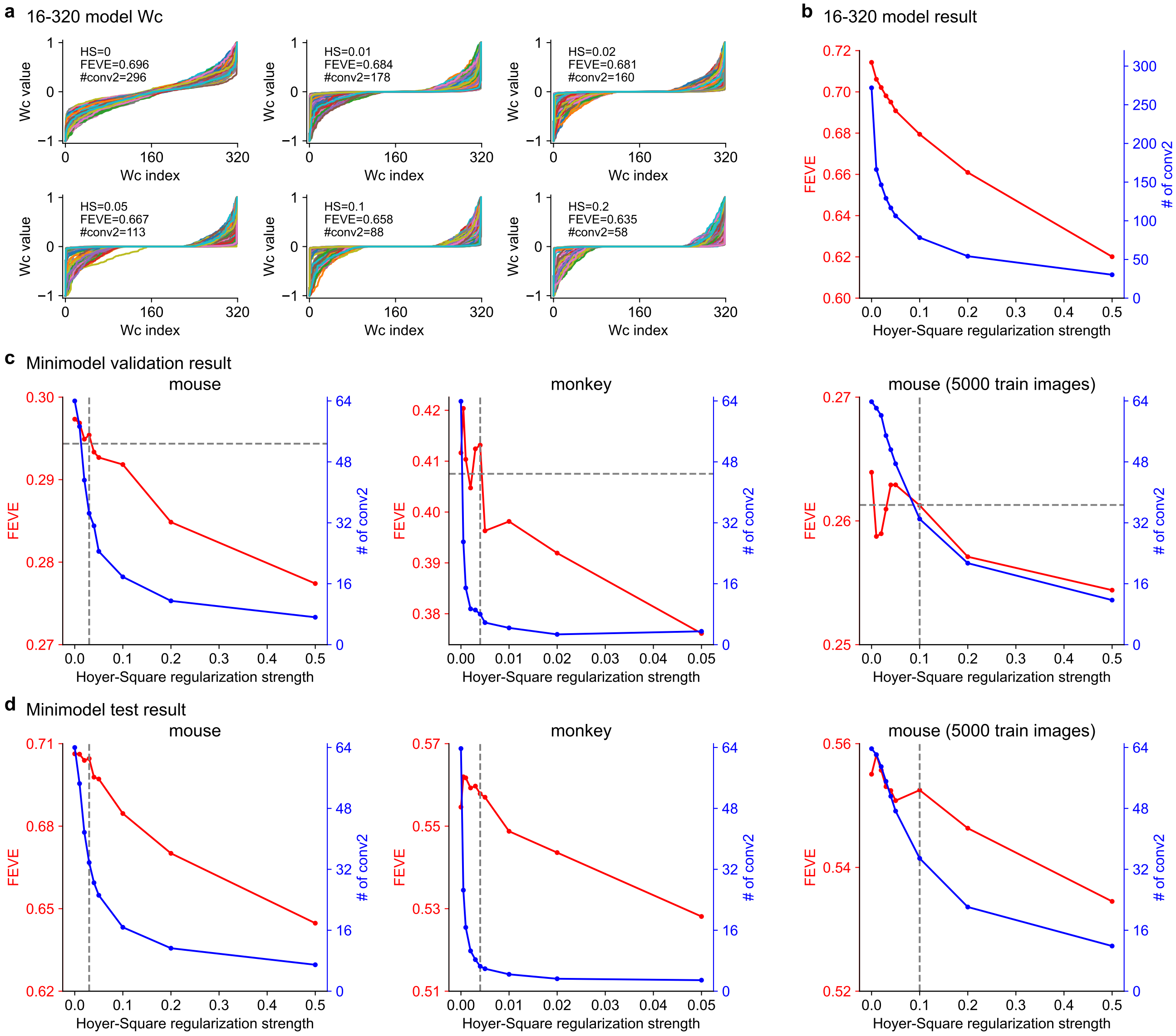

Supplementary Fig. 9: Impact of sparsity regularization on model performance. a, Visualization of $\mathbf{w}_c$ values in the 16-320 model for an example mouse trained with varying strengths of Hoyer-square sparsity regularization. For each neuron, $\mathbf{w}_c$ is a 320-dimensional vector representing the contribution of each conv2 channel to the responses of the neuron. Each panel shows normalized $\mathbf{w}_c$ values of each neuron, average FEVE, and the average number of conv2 channels (#conv2) per neuron for different regularization strengths. **b,** The effect of Hoyer-square regularization strength on the average FEVE and the average number of conv2 channels per neuron in the 16-320 model, averaged across six mice. The FEVE decreased from 0.714 to 0.620 as the average number of conv2 channels per neuron decreased from 272 to 30. **c,** The strength of the Hoyer-square sparsity regularization loss in the minimodel is selected based on the validation performance for models fit to 10 randomly-selected neurons selected from mouse 1 and from the monkey dataset (see Methods). The horizontal dashline shows the 99% of the performance without any regularization. The vertical dash line shows the Hoyer-square sparsity regularization strength selected to train all the minimodels. Minimodels in the first and second plots are trained with the full training set, and the minimodels in the third plot are trained with 5,000 images randomly sampled from the training set. **d,** With 1,000 neurons randomly sampled from all six mice, we show that increasing the strength of the sparsity regularization results in a decrease of performance as well as a decrease in minimodel size. The dashed lines shows the regularization strength used for training all the mouse minimodels in the paper, chosen based on the data in **a**.

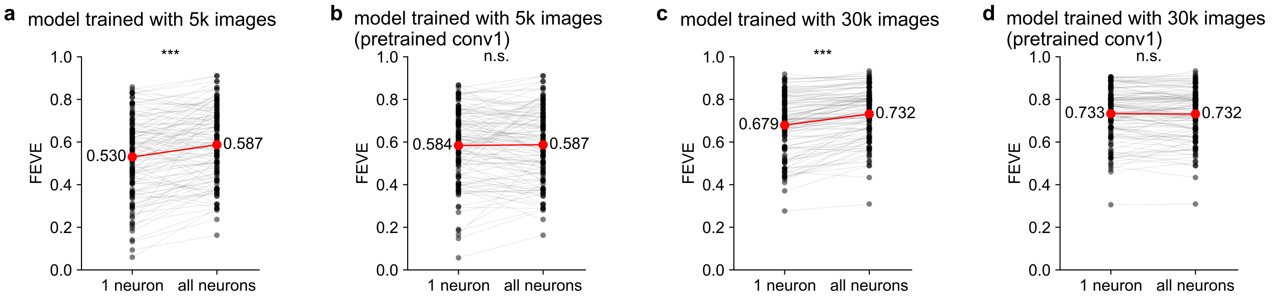

Supplementary Fig. 10: Performance comparison between single-neuron models and population models. We fit the 16-320 model to both individual neurons and the full neural population. FEVE of 120 neurons (20 randomly selected neurons per mouse) was compared between single-neuron models (fit individually) and population models (fit jointly with all other neurons). **a,** Models trained with 5,000 images: FEVE significantly improved when using population models. Each dot represents a single neuron, red line indicates the mean FEVE across neurons (N = 120 neurons, p = 1.06×10^{-6}, paired t-test). **b,** FEVE of neurons fit individually using single-neuron models with conv1 initialized and fixed from the population model, compared to those fit together with all other neurons, trained using 5,000 images (p = 0.76). **c-d,** Same as **a-b**, but with models trained using 30,000 images (p = 9.82×10^{-18}, p = 0.82).

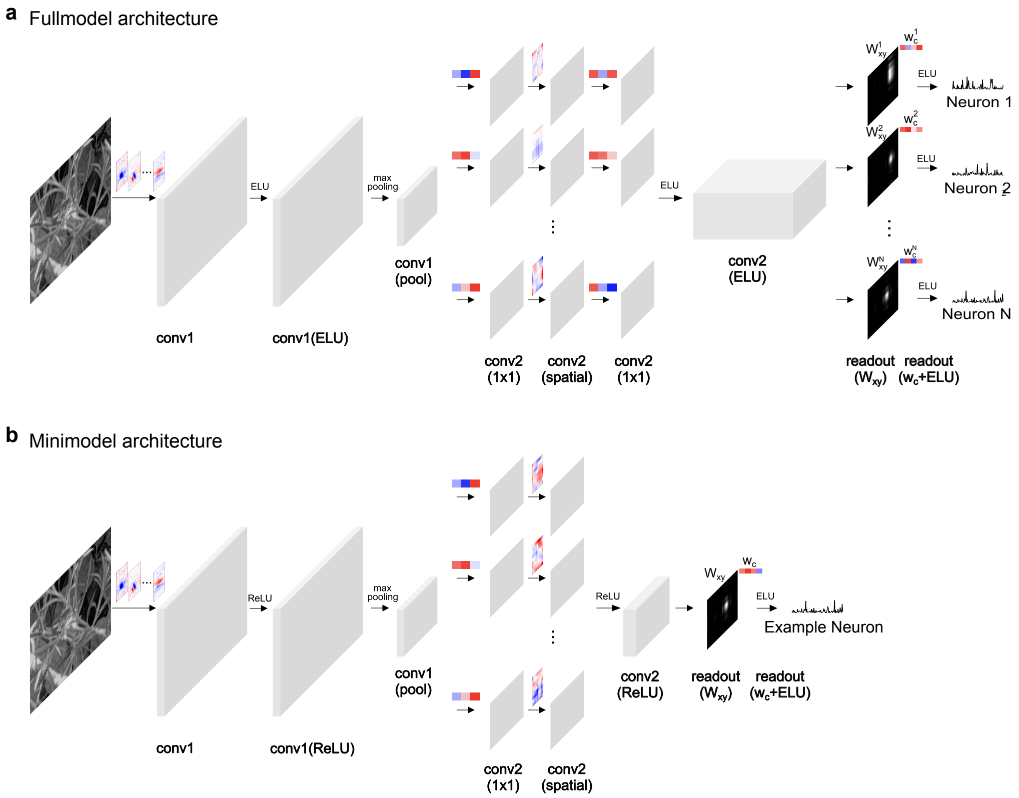

Supplementary Fig. 11: The architecture of the 16-320 model and of the minimodels. a, Detailed structure of the 16-320 model, which comprises two convolutional layers: conv1 and conv2. Each convolutional layer is followed by an ELU activation function. A spatial pooling layer follows conv1. In conv2, depthwise separable convolution is employed, consisting of a 1x1 convolution, a 9x9 spatial convolution, and another 1x1 convolution. The readout layer includes a spatial pooling layer W_{xy} and a channel combination weight $\mathbf{w}_c$. We denote the features after W_{xy} as readout(W_{xy}) and the predictions after $\mathbf{w}_c$ and ELU activation as readout($\mathbf{w}_c$+ELU). **b,** Detailed structure of the minimodels. This model uses ReLU after each convolutional layer instead of ELU. In conv2, the depthwise separable convolution consists of a 1x1 convolution and a 9x9 spatial convolution.

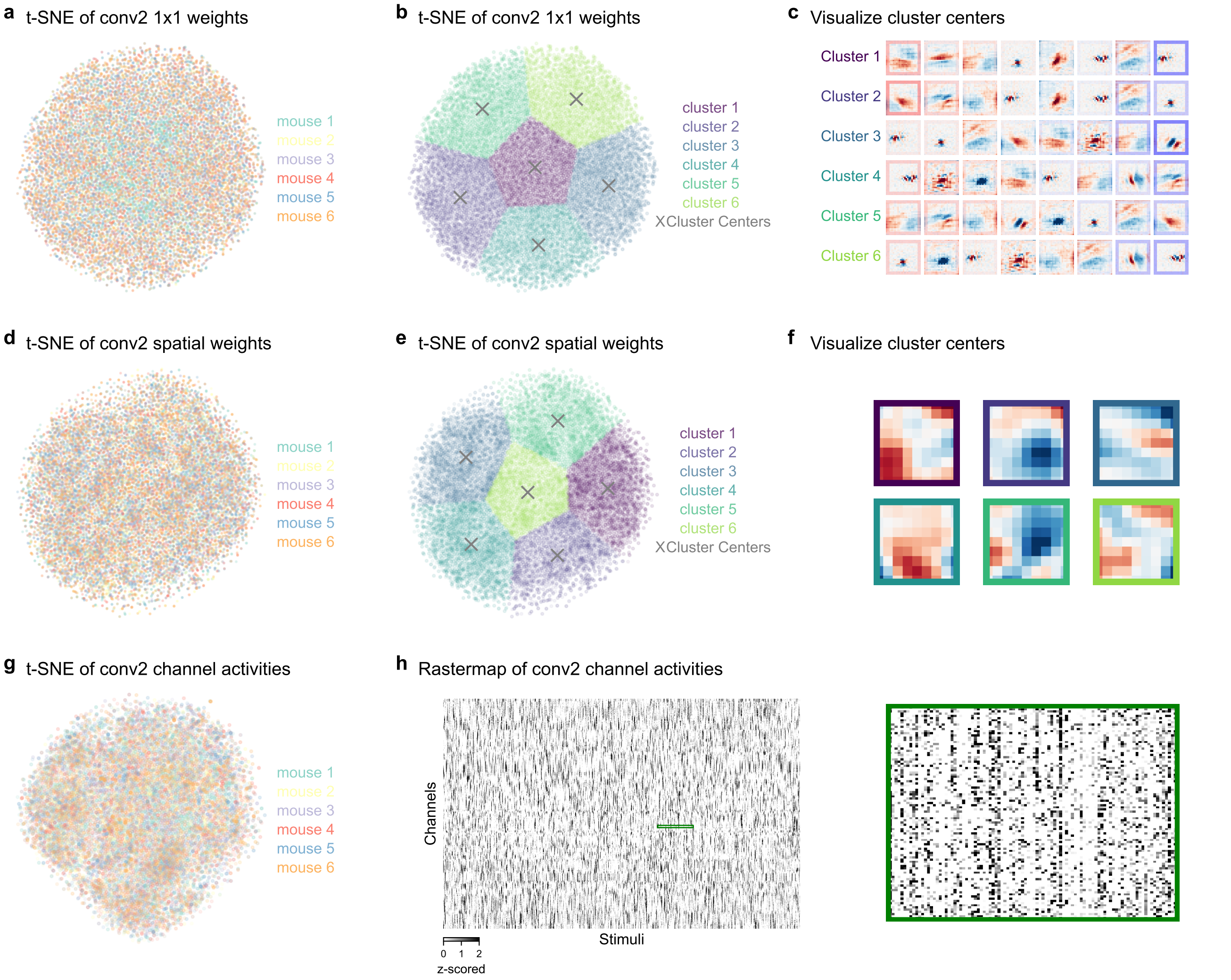

Supplementary Fig. 12: Conv2 layer properties in minimodels. To investigate the properties of conv2 channels in the minimodels, we trained models for 100 randomly selected neurons from each of the six mice using the conv1 weights from mouse 1. All conv2 channels from the 600 neurons were pooled, and only channels with non-zero contribution ($|w_c| > 0$) were included in the analysis. **a,** t-SNE visualization of the 1x1 convolutional weights in the conv2 layer. **b,** K-means clustering of the 1x1 conv weights from **a**. **c,** Visualization of cluster centers from **b**. Each 1x1 convolutional weight represents a 16-dimensional vector corresponding to the contribution of the 16 kernels in conv1. The top 8 contributing kernels for each cluster center are shown (largest $|w_c|$, with color and intensity of the frames indicating their relative contribution based on the 1x1 weights. **d,** t-SNE visualization of the spatial convolutional weights in the conv2 layer. **e,** K-means clustering of the spatial convolutional weights from **d**. **f,** Visualization of cluster centers from **e**. **g,** t-SNE visualization of conv2 channel activities. **h,** Rastermap of conv2 channel responses to 1,000 stimuli, showing activity patterns across channels.

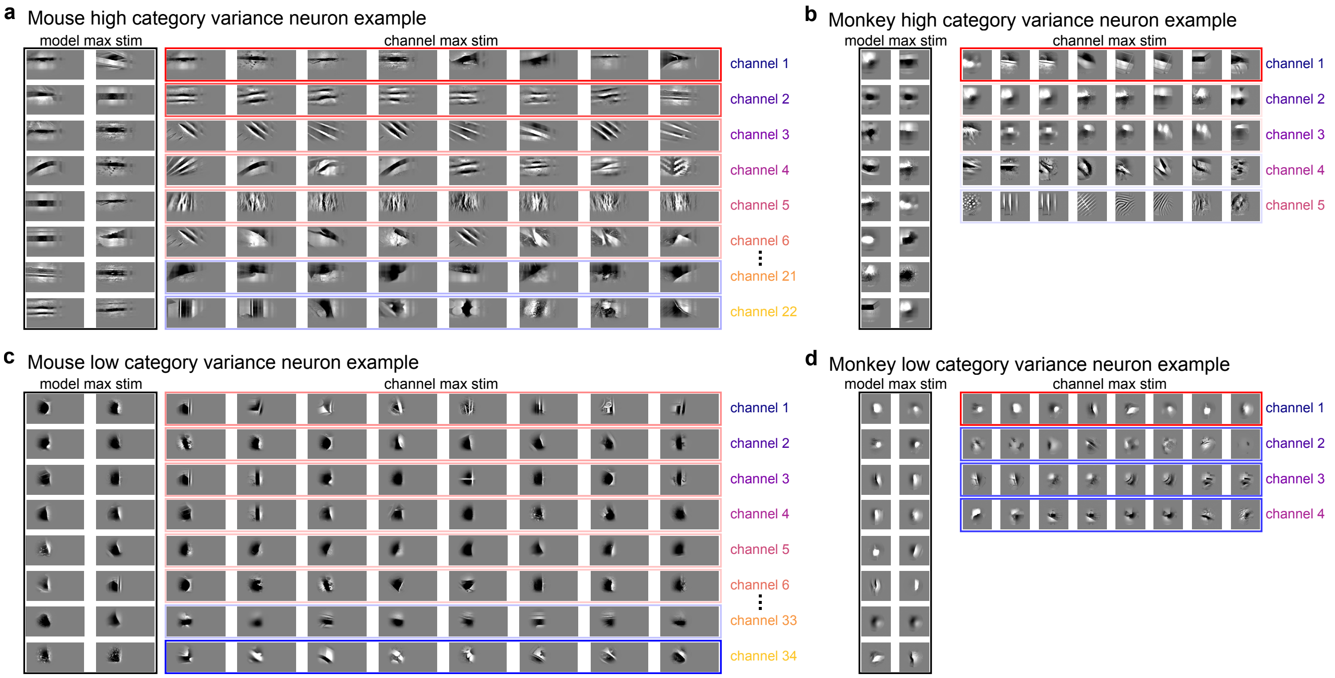

Supplementary Fig. 13: Visualization of neurons with high and low category variance (FECV). a, Maximum stimuli for an example mouse neuron with high category variance (FECV). Left: Top 16 maximum stimuli for the full model output, masked by W_{xy} (see Methods). Right: Top 8 maximum stimuli for the top 6 channels with the largest w_c values and the bottom 2 channels with the smallest w_c channels. The color and intensity indicates the w_c value corresponding to each channel with red signifying positive weights and blue negative weights. **b-d,** Same as **a**, for other example neurons from mouse and monkey.

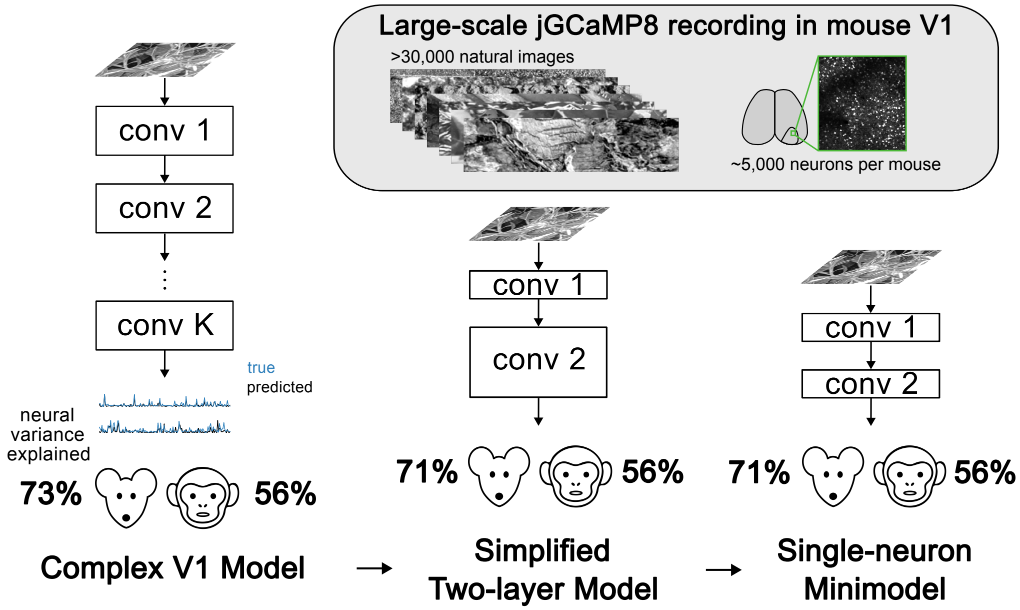

Supplementary Fig. 14: Summary of simplification of mouse and monkey V1 models.
