# Supplementary material for: A simplified minimodel of visual cortical neurons
Source: Nat Commun. 2025 Jul 1;16:5724. doi: 10.1038/s41467-025-61171-9 (PMC12219398; doi:10.1038/s41467-025-61171-9)
Supplement: Supplementary file 2 — Description of Additional Supplementary Files [file 41467_2025_61171_MOESM2_ESM.pdf]

Description of Additional Supplementary Files

File name: Supplementary Movie 1
Description: Visual stimulus presentation and simultaneous neural imaging. Top: presented natural images. Bottom: raw calcium imaging data from mouse V1 across four imaging depths recorded at a rate of 30 Hz. Video is shown at 0.5x speed.
